# Validity of a Semi-Quantitative Food Frequency Questionnaire for Collegiate Athletes

**DOI:** 10.2188/jea.JE20150104

**Published:** 2016-06-05

**Authors:** Ayaka Sunami, Kazuto Sasaki, Yoshio Suzuki, Nobuhide Oguma, Junko Ishihara, Ayumi Nakai, Jun Yasuda, Yuri Yokoyama, Takahiro Yoshizaki, Yuki Tada, Azumi Hida, Yukari Kawano

**Affiliations:** 1 Department of Food and Nutritional Science, Graduate School of Tokyo University of Agriculture, Tokyo, Japan; 1 東京農業大学大学院 農学研究科 食品栄養学専攻; 2 Research Fellow of the Japan Society for the Promotion of Science, Tokyo, Japan; 2 日本学術振興会特別研究員; 3 Graduate School of Health and Sports Science, Juntendo University, Inzai, Chiba, Japan; 3 順天堂大学大学院 スポーツ健康科学研究科; 4 Faculty of Nutritional Science, Sagami Women’s University, Sagamihara, Kanagawa, Japan; 4 相模女大学 栄養科学部 管理栄養学科; 5 Department of Nutritional Science, Toyo University, Ora-gun, Gunma, Japan; 5 東洋大学 食環境科学部; 6 Faculty of Food and Nutritional Science, Tokyo University of Agriculture, Tokyo, Japan; 6 東京農業大学 応用生物科学部 栄養科学科

**Keywords:** dietary assessment, athlete, FFQ, 24-h dietary recall, 食事調査, アスリート, FFQ, 24時間思い出し法

## Abstract

**Background:**

Food frequency questionnaires (FFQs) have been developed and validated for various populations. To our knowledge, however, no FFQ has been validated for young athletes. Here, we investigated whether an FFQ that was developed and validated to estimate dietary intake in middle-aged persons was also valid for estimating that in young athletes.

**Methods:**

We applied an FFQ that had been developed for the Japan Public Health Center-based Prospective Cohort Study with modification to the duration of recollection. A total of 156 participants (92 males) completed the FFQ and a 3-day non-consecutive 24-hour dietary recall (24hDR). Validity of the mean estimates was evaluated by calculating the percentage differences between the 24hDR and FFQ. Ranking estimation was validated using Spearman’s correlation coefficient (CC), and the degree of miscategorization was determined by joint classification.

**Results:**

The FFQ underestimated energy intake by approximately 10% for both males and females. For 35 nutrients, the median (range) deattenuated CC was 0.30 (0.10 to 0.57) for males and 0.32 (−0.08 to 0.62) for females. For 19 food groups, the median (range) deattenuated CC was 0.32 (0.17 to 0.72) for males and 0.34 (−0.11 to 0.58) for females. For both nutrient and food group intakes, cross-classification analysis indicated extreme miscategorization rates of 3% to 5%.

**Conclusions:**

An FFQ developed and validated for middle-aged persons had comparable validity among young athletes. This FFQ might be useful for assessing habitual dietary intake in collegiate athletes, especially for calcium, vitamin C, vegetables, fruits, and milk and dairy products.

## 要約

**背景:**

食物摂取頻度調査票（food frequency questionnaires、FFQ）は様々な集団を対象に開発され、妥当性が検証されている。しかし我々の知る限りでは、若年アスリートのためのFFQの妥当性はいまだ検証されていない。そこで我々は、中高年者の食事量を推定するために開発され、妥当性が検証されているFFQが若年アスリートの食事量推定においても妥当と認められるか否か、検討した。

**目的:**

本研究では「多目的コホートに基づくがん予防など健康の維持・増進に役立つエビデンスの構築に関する研究」のために開発されたFFQを、思い出し期間を変更して用いた。156名（うち、男性92名）がFFQおよび不連続3日間の24時間思い出し食事調査（24-hour dietary recall 、24hDR）を完了した。平均値推定の妥当性は、24hDRとFFQの%差を用いて評価した。順位推定の妥当性には、スピアマンの順位相関係数（correlation coefficient、CC）および五分位の同時分類による一致度を用いた。

**結果:**

男女ともに、FFQはエネルギー摂取量をおよそ10%過小評価した。栄養素35項目では、deattenuated CCの中央値（範囲）が男性で0.30（0.10から0.57）、女性で0.32（-0.08から0.62）であった。食品群19項目では、男性で0.32（0.17から0.72）、女性で0.34（-0.11から0.58）であった。栄養素および食品群で正反対の分位に分類された割合は3%から5%であった。

**結論:**

中高年者の食事量推定のために開発され、妥当と評価されたFFQは、若年アスリートにおいても同等の妥当性を示した。今回のFFQは大学アスリートの習慣的食事量を評価するには有用で、特に栄養素ではカルシウム、ビタミンC、食品群では野菜類、果実類、牛乳・乳製品の評価に優れているだろう。

## INTRODUCTION

Adequate dietary intake is an important factor in the ability of athletes to maintain an optimum physical condition and train effectively. The International Olympic Committee released a statement on sports nutrition in 2010^1^ and nutritional intake guidelines for athletes^2^^–^^6^ in 2011. Despite the global availability of these guidelines, however, discussion in Japan on the establishment of a survey to easily and rapidly assess the dietary intake of athletes has been insufficient.

Common dietary survey methods include the dietary record method (DRM), 24-hour dietary recall (24hDR),^7^ and the food frequency questionnaire (FFQ).^8^ When conducting a dietary survey, the strengths and weaknesses of each survey method should be considered to ensure that the most appropriate one is selected. Although the DRM and 24hDR are commonly used as reference methods, they place a large burden on participants, and calculating dietary intake is expensive in terms of labor, time, and cost. In contrast, FFQs are a simple and inexpensive method that places minimal burden on participants.

Although athletes require rapid feedback, the number of dieticians available to meet this demand is limited. We therefore consider the FFQ to be a candidate survey method for assessment of the habitual dietary intake of athletes.^9^ Although FFQs have been developed and validated for various populations in Japan,^10^ we are unaware of the validation of any FFQ for athletes.

Here, we investigated whether an FFQ that was developed and validated to estimate the dietary intake of middle-aged persons was also valid in estimating the intake of young athletes.

## METHODS

### Participants

Participants were 171 college students belonging to the soccer, basketball, track and field, handball, tennis, judo, or volleyball sports clubs of various universities around the Tokyo metropolitan area. Prior to the study, the purpose and methodology of the study were explained orally and in writing to the participants, and written consent was obtained. The study was conducted following approval from the Ethics Committee of the Tokyo University of Agriculture (Approval No. 1124) and the Ethics Committee of Juntendo University Graduate School of Health and Sports Science (No. 25-47).

### Study design

**Figure 1.**
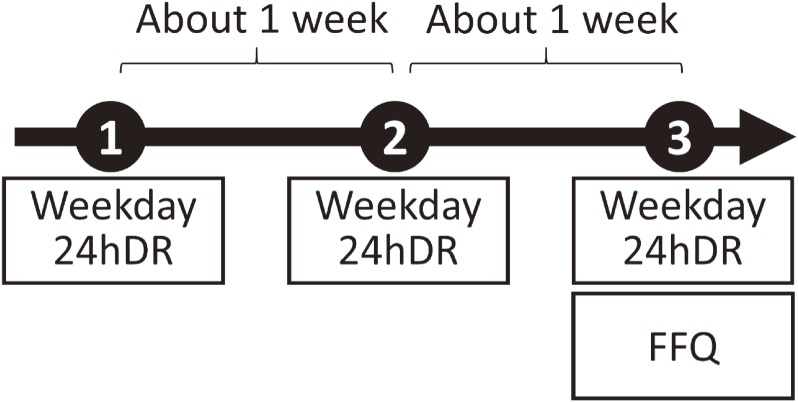
Study design 24hDR, 24-hour dietary recall; FFQ, food frequency questionnaire.

The study was conducted for approximately 2 months between the end of August and the beginning of October 2013. The study design is shown in Figure 1. Initially, the 24hDRs were conducted on three weekdays, with an interval of approximately 1 week between each. After completion of the third 24hDR, a dietary survey was then immediately conducted using the FFQ.

### FFQ

Following an explanation of how to complete the self-administered FFQ, participants were assembled together and asked to complete the FFQ themselves. During self-administration, participants were shown pictures of food items listed in the FFQ using a projector as a guide to help them estimate their approximate food intake. After completion, a registered dietitian reviewed the records for completeness and accuracy.

The FFQ was developed for a middle-aged population in the Japan Public Health Center-based Prospective Cohort Study and contained 138 food and 20 beverage items and 14 questions regarding seasonings.^11^ The FFQ included questions for each food item regarding the frequency of consumption and portion size for each food item. Frequency was selected from a 9-level scale, which ranged from ‘less than once per month’ to ‘7 times or more per day’. Portion size was selected from a 3-level scale, as follows: ‘smaller than a standard portion size (≤0.5 times)’, ‘same as a standard portion size’, or ‘larger than the standard portion size (>1.5 times)’. For beverages, frequency was selected from a 9-level scale, which ranged from ‘less than once a week’ to ‘10 drinks or more everyday’, and portion size was not queried. In the present study, the duration of recollection of the FFQ^11^ was modified from 1 year to 1 month.

In accordance with the original FFQ, we used the Standard Tables of Food Composition in the Japan Fifth Revised and Enlarged Edition (Food Composition Table)^12^ to calculate the intake of energy and nutrients. In calculating the intakes of certain food groups, ice cream was listed as a confectionery to emphasize the perspective of dietary education. Ice cream is categorized in ‘milk and dairy products’ in the original FFQ but is listed under ‘confectioneries’ in the ‘Japanese Food Guide Spinning Top’ developed by the Japanese government.^13^ Ice cream has consistently ranked as the second most frequently consumed food among ‘confectioneries’ consumed by female collegiate athletes.^14^

### 24hDR

Participant interviews were conducted in person by registered dietitians or by well-trained university students enrolled in a registered dietitian and nutritionist course. The participants were asked to recall all foods and beverages consumed within the last 24 hours, as well as their activities during that period. Prior to the survey, all personnel received training for conducting interviews using standardized recall tools. Based on the multiple-pass interview technique,^15^^–^^17^ the procedure for recall was divided into four steps. In step 1, participants were asked to recall the situations, time, and details of any food or beverages consumed, along with details of their daily activities. In step 2, participants were asked whether the food and beverages consumed were prepared at home (homemade food), prepared at a restaurant (dine-out food), or bought at a supermarket or convenience store (take-out food). For homemade food, ingredients were documented in detail. For dine-out food, the food and beverage items consumed and the name of the restaurant and of the dish as written in the menu were recorded. For take-out food, in addition to the consumed food and beverage items, the name of the dish or product along with those of the manufacturer and retailer were recorded. In step 3, the weight of the food was documented. In step 4, based on the name of the dish and preparation method, the interviewers re-checked whether food items that were possibly forgotten were included in the meals, with particular focus on preparation methods and seasonings (eg, whether butter or margarine was used on toast or not) and details of each food category (eg, regular or low-fat milk). The amount of intake for each food item was also recorded. Finally, a registered dietitian checked the forms to ensure that they had been completed with no missing information. 24hDR interviews lasted an average of 32 min per person.

From the completed 24hDR, the intakes of energy, nutrients, and food groups consumed each day were calculated using Excel Eiyokun Version 6.0 (Kenpakusha, Tokyo, Japan), which is based on the Food Composition Table.^12^ Nutrient intakes from supplements were not included in these calculations. For dine-out and take-out foods, the name and weight of each food item was determined using the product label and food photography along with the 24hDR data. When details were unclear, the manufacturer was contacted directly for clarification. Food groups were categorized according to the FFQ. Once the survey was completed, the results of the analysis of the 24hDR were returned to the participants along with comments from the registered dietitian.

### Statistical analysis

Statistical analysis was performed for the 156 participants (92 males) who fully completed the 3-day 24hDR and FFQ. The mean of the three 24hDRs was used to obtain a reference level of food intake.

**Figure 2.**
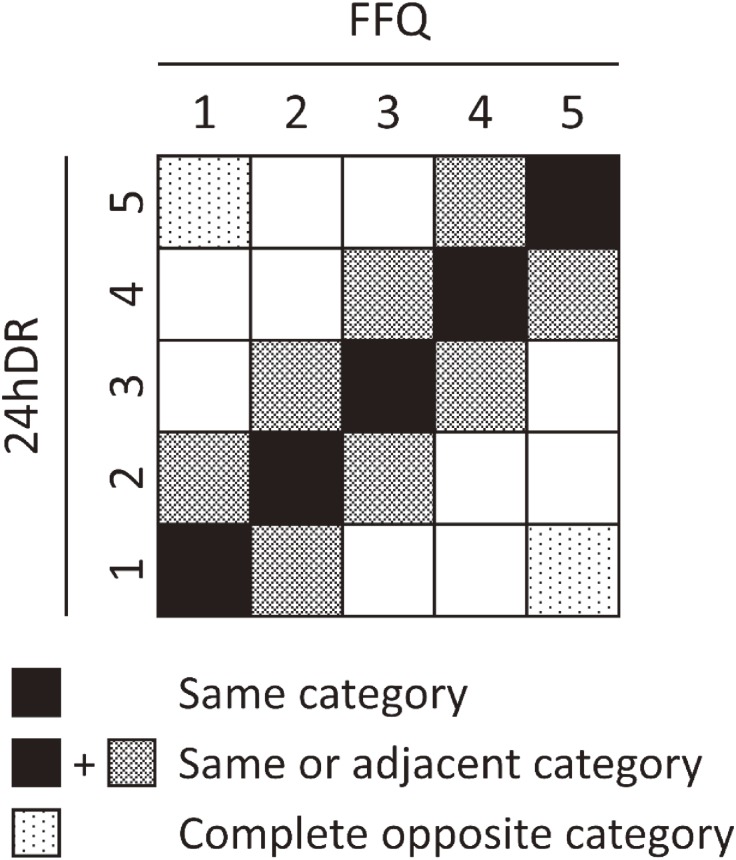
Joint classification method 24hDR, 24-hour dietary recall; FFQ, food frequency questionnaire.

The ranking validity of the FFQ was examined by calculating Spearman’s rank correlation coefficient (CC) between the 24hDR and FFQ. CCs were calculated for both the raw and energy-adjusted intake data. For energy adjustment by the residual method, the raw data were converted logarithmically. As CCs for 24hDRs and FFQs are affected and attenuated by the fluctuations of daily intake, a number of studies have used Rosner et al’s^18^ method of statistical correction of CC.^11^^,^^19^^,^^20^ The ratio between inter- and intra-individual variance in 24hDR was therefore calculated and applied to equation (1) to correct the CC, as follows:

$${\text{Deattenuated CC}}_{\text{x}}={\text{en-CC}}_{\text{x}}\times \sqrt{1+\lambda_{\text{x}}/n}$$ (1)

where en-CC_x_ denotes the CC of energy-adjusted nutrient x, *λ*_x_ denotes the ratio of inter-individual variance to the intra-individual variance for 24hDR, and *n* denotes the number of surveyed recalls (*n* = 3 in the present study) for 24hDR. A review of FFQ validity studies targeting Japanese populations established validity at the following levels: high validity (median CC ≥0.6), moderate validity (0.40–0.59), fair validity (0.30–0.39), and poor validity (<0.3).^10^ Based on these previous findings, items with CCs that exceeded 0.3 were considered to exhibit ranking validity in the present study. To assess the validity of categorization, the energy-adjusted intake from the 24hDR and FFQ was divided into quintiles, and the percentages of participants classified into the same, same or adjacent, and completely opposite categories were calculated by the joint classification method (Figure 2).

The difference in mean values between the 24hDR and FFQ was calculated as a percentage from the following equation (2):

% difference=(FFQ−24hDR)/24hDR×100 (2)

Values were expressed as either the mean value (standard deviation), median (range), or as the number of people (%). All statistical analyses were done using IBM SPSS Statistics ver. 22 (IBM Japan, Ltd., Tokyo, Japan).

## RESULTS

### Characteristics of study population

The study population and their affiliated sports clubs are characterized in Table 1. Of the total study population, more than 80% lived either in dormitories or alone, and 20% lived with their families.

**Table 1.** Characteristics of the participants

	Male (*n* = 92)	Female (*n* = 64)
Height, cm	174.7 (6.5)	163.4 (5.8)
Weight, kg	68.7 (8.4)	56.1 (5.9)
Body mass index, kg/m^2^	22.5 (2.1)	21.0 (1.5)
Exercise frequency, days/week	5.7 (0.9)	5.8 (0.6)
Exercise duration, hours/day	2.9 (0.9)	3.8 (1.1)

Residence condition		
Parents’ house	15 (16)	5 (8)
Dormitory	52 (57)	33 (52)
Alone	25 (27)	26 (41)

Club		
Soccer	32 (35)	10 (16)
Basketball	20 (22)	17 (27)
Track and Field	20 (22)	14 (22)
Handball	10 (11)	8 (13)
Tennis	5 (5)	5 (8)
Judo	5 (5)	—
Volleyball	—	10 (16)

Data are presented as mean (standard deviation) or number (percent).

### Validity of mean intake and FFQ

The data for nutrient intake are shown in Table 2. Differences of −9% in males and −10% in females were observed for energy intake, and the majority of nutrients were within a ±20% range in both males and females. The largest difference observed was for retinol, at 77% for males and 32% for females. For crude CCs, the median CC was 0.29 for males (from 0.14 for water-soluble dietary fiber to 0.43 for calcium) and 0.36 for females (from 0.15 for polyunsaturated fatty acids to 0.55 for folic acid). The median energy-adjusted CC was 0.27 (from 0.10 for zinc to 0.53 for calcium) for males and 0.29 for females (from −0.08 for total fat to 0.56 for vitamin K). The median deattenuated CC was 0.30 (from 0.10 for zinc to 0.57 for calcium) for males and 0.32 for females (from −0.08 for total fat to 0.62 for vitamin K).

**Table 2.** Energy and nutrient intakes from 3-day non-consecutive 24hDR and FFQ, percentage difference between 24hDR and FFQ, and their correlations in males and females

	Male (*n* = 92)									Female (*n* = 64)								
	
	24hDR		FFQ		%^a^	Correlation coefficient^b^				24hDR		FFQ		%^a^	Correlation coefficient^b^			
	
						Crude	Energy-adjusted	Deattenuated^c^	Reference^d^						Crude	Energy-adjusted	Deattenuated^c^	Reference^d^

	Mean	SD	Mean	SD						Mean	SD	Mean	SD					
Energy, kcal	3184	940	2906	1194	−9	0.34*	—	0.36	0.53	2142	506	1919	602	−10	0.32*	—	0.34	0.34
Protein, g	100.1	32.5	82.0	35.5	−18	0.24*	0.24*	0.26	0.67	65.5	16.4	59.0	22.8	−10	0.36*	0.19	0.20	0.47
Total fat, g	83.7	34.2	76.7	40.6	−8	0.18	0.13	0.14	0.42	64.0	18.6	57.1	28.6	−11	0.22	−0.08	−0.08	0.35
SFA, g	24.93	11.01	24.71	13.38	−1	0.19	0.17	0.18	0.44	19.76	6.48	18.24	9.53	−8	0.31*	0.02	0.02	0.41
MUFA, g	31.23	14.43	29.40	16.46	−6	0.19	0.15	0.17	0.38	22.72	7.35	21.16	11.53	−7	0.22	−0.07	−0.07	0.49
PUFA, g	16.57	6.34	14.41	7.43	−13	0.22*	0.22*	0.24	0.72	12.69	4.12	11.25	5.60	−11	0.15	0.17	0.19	0.38
Cholesterol, mg	390	200	286	183	−27	0.29*	0.22*	0.24	0.51	317	133	260	181	−18	0.31*	0.38*	0.42	0.38
Carbohydrate, g	486.6	152.9	452.4	182.8	−7	0.37*	0.21*	0.22	0.56	319.9	81.8	286.2	76.0	−11	0.26*	−0.01	−0.01	0.43

Sodium, mg	4461	1267	3769	1894	−16	0.23*	0.31*	0.34	0.45	3359	1181	2603	1403	−23	0.23	0.42*	0.47	0.47
Salt Eq, g	11.2	3.2	9.5	4.8	−16	0.23*	0.30*	0.33	0.42	8.4	3.0	6.5	3.5	−23	0.23	0.42*	0.47	0.46
Potassium, mg	2594	840	2601	1262	0	0.34*	0.43*	0.46	0.65	2470	724	2222	957	−10	0.48*	0.45*	0.48	0.70
Calcium, mg	564	249	581	365	3	0.43*	0.53*	0.57	0.64	534	219	503	239	−6	0.34*	0.40*	0.43	0.61
Magnesium, mg	275	77	276	120	0	0.30*	0.52*	0.55	0.58	247	67	215	83	−13	0.42*	0.54*	0.59	0.54
Phosphorus, mg	1275	394	1191	524	−7	0.31*	0.48*	0.51	0.65	993	267	897	348	−10	0.38*	0.34*	0.37	0.47
Iron, mg	8.0	2.4	7.3	3.4	−8	0.19	0.26*	0.28	0.68	7.2	2.1	6.2	2.8	−14	0.38*	0.41*	0.44	0.55
Zinc, mg	12.8	4.2	11.3	4.9	−12	0.30*	0.10	0.10	0.65	8.7	2.3	7.3	2.5	−16	0.31*	0.25*	0.27	0.34
Copper, mg	1.55	0.47	1.48	0.61	−5	0.32*	0.36*	0.38	0.74	1.21	0.35	1.08	0.35	−11	0.46*	0.55*	0.59	0.49
Manganese, mg	4.22	1.47	4.14	2.38	−2	0.34*	0.17	0.18	0.44	3.07	1.20	2.69	1.00	−12	0.44*	0.26*	0.28	0.41

Retinol, µg	197	110	349	444	77	0.31*	0.24*	0.27	0.56	204	221	269	339	32	0.31*	0.12	0.14	0.16
Retinol Eq, µg	430	241	576	516	34	0.26*	0.20	0.21	0.23	422	243	505	416	19	0.30*	0.14	0.17	0.33
β-carotene Eq, µg	2751	2534	2710	2036	−1	0.30*	0.36*	0.39	0.52	2593	1422	2799	1781	8	0.25*	0.24	0.27	0.62
Vitamin D, µg	5.4	5.2	4.0	2.6	−27	0.30*	0.29*	0.33	0.88	3.8	3.3	3.8	3.2	1	0.41*	0.22	0.25	0.37
α-tocopherol, mg	7.7	2.9	6.8	3.4	−12	0.17	0.22*	0.24	0.48	7.6	3.0	6.7	3.8	−13	0.43*	0.45*	0.48	0.51
Vitamin K, µg	199	105	200	123	0	0.41*	0.27*	0.30	0.79	190	103	180	125	−5	0.54*	0.56*	0.62	0.94
Vitamin B_1_, mg	1.19	0.44	1.05	0.52	−12	0.24*	0.28*	0.30	0.54	0.96	0.28	0.84	0.38	−12	0.40*	0.24	0.26	0.42
Vitamin B_2_, mg	1.44	0.48	1.33	0.72	−7	0.33*	0.48*	0.52	0.42	1.25	0.38	1.10	0.48	−12	0.39*	0.29*	0.31	0.53
Niacin, mg	19.3	7.0	17.0	8.8	−11	0.19	0.17	0.18	0.44	12.7	3.9	11.9	6.3	−6	0.41*	−0.03	−0.04	0.32
Vitamin B_6_, mg	1.39	0.49	1.22	0.60	−12	0.27*	0.19	0.21	0.44	1.09	0.34	0.97	0.46	−11	0.51*	0.32*	0.35	0.57
Vitamin B_12_, µg	5.8	3.8	5.2	2.9	−10	0.42*	0.49*	0.53	0.57	3.9	2.9	4.0	3.3	3	0.37*	0.09	0.10	0.67
Folate, µg	291	103	277	146	−5	0.38*	0.25*	0.27	0.66	312	97	255	122	−18	0.55*	0.48*	0.54	0.41
Pantothenic acid, mg	8.05	2.59	7.80	3.48	−3	0.33*	0.41*	0.43	0.67	6.34	1.67	5.98	2.28	−6	0.38*	0.29*	0.32	0.66
Vitamin C, mg	130	98	128	104	−2	0.26*	0.37*	0.41	0.73	163	85	135	87	−17	0.48*	0.49*	0.55	0.51

Total dietary fiber, g	13.0	3.5	12.2	5.7	−6	0.27*	0.31*	0.34	0.67	12.2	3.6	9.7	4.0	−20	0.32*	0.41*	0.45	0.53
Water soluble, g	3.2	1.1	2.9	1.6	−10	0.14	0.18	0.20	0.65	3.3	0.9	2.5	1.2	−24	0.31*	0.31*	0.35	0.56
Water insoluble, g	9.1	2.5	8.7	3.9	−5	0.26*	0.28*	0.31	0.71	8.3	2.6	6.9	2.6	−17	0.26*	0.28*	0.31	0.44

**Median**						**0.29**	**0.27**	**0.30**	**0.57**						**0.36**	**0.29**	**0.32**	**0.47**

24hDR, 24-hour dietary recall; Eq, equivalent; FFQ, food frequency questionnaire; MUFA, monounsaturated fatty acid; PUFA, polyunsaturated fatty acid; SD, standard deviation; SFA, saturated fatty acid.

^a^Percentage differences: (FFQ − 24hDR)/24hDR * 100 (%); ^b^Spearman’s correlation coefficients between 24hDR and FFQ; *Significant correlation coefficients (*P* < 0.05). ^c^Deattenuated CC_x_ = observed CC_x_ * SQRT (1 + *λ*_x_/*n*), where *λ*_x_ is the ratio of within- to between-individual variance for nutrient *x*, and *n* is number of 24hDR; observed CCs were based on energy-adjusted values except for energy intake; ^d^Deattenuated CC among middle-aged population (reference number ^11^).

Intake data for each food group are shown in Table 3. Lower percentage differences in both males and females were observed for the following food items: ‘cereals’ (1% in males and 10% in females), ‘vegetables’ (9% in males and 4% in females), and ‘fungi’ (5% in males and 9% in females). Larger differences in both males and females were observed for the following food items: ‘sugar’ (−94% in males and −96% in females), ‘beverages’ (−45% in males and −54% in females), and ‘seasonings and spices’ (−65% in males and −72% in females). Regarding crude CCs, the median CC was 0.33 for males (from 0.04 in ‘sugar’ to 0.57 in ‘milk and dairy products’) and 0.34 for females (from −0.01 in ‘sugar’ to 0.59 in ‘milk and dairy products’). The median energy-adjusted CC was 0.30 (from 0.16 for ‘sugar’ to 0.67 for ‘milk and dairy products’) for males and 0.31 (from −0.10 in ‘sugar’ to 0.56 in ‘milk and dairy products’) for females. The median deattenuated CC was 0.32 for males (from 0.17 for ‘sugar’ to 0.72 for ‘milk and dairy products’) and 0.34 for females (from −0.11 for ‘sugar’ to 0.58 for ‘milk and dairy products’).

**Table 3.** Food group intakes from 3-day non-consecutive 24hDR and FFQ, percentage difference between 24hDR and FFQ, and their correlations in males and females

	Male (*n* = 92)									Female (*n* = 64)								
	
	24hDR		FFQ		%^a^	Correlation coefficient^b^				24hDR		FFQ		%^a^	Correlation coefficient^b^			
	
						Crude	Energy- adjusted	Deattenuated^c^	Reference^d^						Crude	Energy- adjusted	Deattenuated^c^	Reference^d^

	Mean	SD	Mean	SD						Mean	SD	Mean	SD					
Cereals	871.5	302.7	880.8	395.1	1	0.34*	0.27*	0.28	0.51	436.1	142.0	477.7	128.2	10	0.50*	0.46*	0.49	0.41
Potatoes and starches	24.1	37.3	19.5	17.6	−19	0.21*	0.18	0.20	0.49	25.4	29.7	18.1	15.8	−29	0.37*	0.35*	0.40	0.39
Sugar	9.0	7.6	0.6	1.3	−94	0.04	0.16	0.17	0.30	8.8	6.0	0.4	0.8	−96	−0.01	−0.10	−0.11	0.07
Pulses	30.2	55.5	45.6	41.6	51	0.35*	0.30*	0.31	0.66	65.8	97.1	82.0	106.9	25	0.48*	0.51*	0.54	0.45
Nuts and seeds	1.4	2.2	1.0	1.8	−30	0.34*	0.28*	0.32	0.40	2.7	4.2	0.5	0.7	−82	−0.01	0.00	0.00	−0.09
Vegetables	149.8	83.4	162.6	125.8	9	0.35*	0.37*	0.40	0.55	156.8	72.7	163.1	110.1	4	0.38*	0.38*	0.42	0.52
Green and yellow vegetables	42.4	45.0	75.3	74.8	78	0.39*	0.43*	0.47	0.59	55.4	36.7	77.8	57.1	40	0.34*	0.26*	0.29	0.57
White vegetables	107.4	54.8	87.3	65.6	−19	0.27*	0.28*	0.30	0.68	101.4	53.9	85.3	68.7	−16	0.31*	0.40*	0.45	0.57
Fruits	209.8	245.9	344.6	341.3	64	0.45*	0.45*	0.48	0.69	295.6	205.1	353.1	266.0	19	0.44*	0.48*	0.52	0.63
Fungi	2.8	4.6	2.9	5.2	5	0.36*	0.30*	0.34	0.57	3.5	4.3	3.2	3.6	−9	0.18	0.14	0.17	0.46
Algae	5.6	5.0	6.4	7.1	13	0.33*	0.34*	0.37	0.22	6.4	8.2	3.1	4.7	−51	0.41*	0.50*	0.57	0.47
Fish and shellfish	45.8	46.5	30.4	21.3	−34	0.33*	0.36*	0.40	0.69	25.5	22.3	28.5	27.9	12	0.37*	0.29*	0.34	0.57
Meats	160.2	88.5	136.6	96.0	−15	0.21*	0.21*	0.23	0.70	88.9	46.4	77.0	58.3	−13	0.24	0.02	0.02	0.36
Eggs	37.0	34.2	23.2	28.7	−37	0.35*	0.33*	0.36	0.67	36.8	25.6	30.2	36.1	−18	0.45*	0.43*	0.49	0.53
Milk and dairy products	148.4	146.4	234.3	257.5	58	0.57*	0.67*	0.72	0.66	133.8	163.8	191.4	167.6	43	0.59*	0.56*	0.58	0.76
Fats and oils	17.5	9.0	12.6	7.6	−28	0.16	0.19	0.21	0.45	11.6	6.6	9.5	6.2	−18	0.23	0.13	0.14	0.73
Confectioneries	66.9	68.5	61.1	58.7	−9	0.27*	0.26*	0.29	0.45	91.6	64.8	54.2	40.4	−41	0.30*	0.31*	0.34	0.43
Beverages	1344.9	782.7	743.3	722.3	−45	0.22*	0.38*	0.40	0.40	1058.8	624.4	482.8	291.9	−54	0.10	0.14	0.15	0.35
Seasonings and spices	63.2	26.7	22.4	14.9	−65	0.29*	0.28*	0.31	0.10	53.5	23.4	14.8	13.5	−72	0.15	0.15	0.16	−0.36

**Median**						**0.33**	**0.30**	**0.32**	**0.55**						**0.34**	**0.31**	**0.34**	**0.46**

24hDR, 24-hour dietary recall; FFQ, food frequency questionnaire; SD, standard deviation.

Food group intakes are reported in grams.

^a^Percentage differences: (FFQ − 24hDR)/24hDR * 100 (%); ^b^Spearman’s correlation coefficients between 24hDR and FFQ; *Significant correlation coefficients (*P* < 0.05). ^c^Deattenuated CC_x_ = observed CC_x_ * SQRT (1 + *λ*_x_/*n*), where *λ*_x_ is the ratio of within- to between-individual variance for nutrient x, and *n* is number of 24hDR; observed CCs were based on energy-adjusted values except for energy intake; ^d^Deattenuated CC among middle-aged population (reference number ^11^).

Table 4 shows the degree of coincidence for nutrient intakes. Median percentages were 26% for males and 28% for females for the ‘same category’, 61% for males and 63% for females for the ‘same or adjacent category’, and 3% for males and 5% for females for the ‘completely opposite category’.

**Table 4.** Comparison of FFQ with 3-day non-consecutive 24hDR for energy-adjusted nutrients based on joint classification by quintile (%)

	Males (*n* = 92)			Females (*n* = 64)		
	
	Same category	Same or adjacent category	Complete opposite category	Same category	Same or adjacent category	Complete opposite category
Energy^a^	35	70	5	28	63	2
Protein	26	59	5	28	58	3
Total fat	24	55	8	19	47	9
SFA	21	58	4	19	47	6
MUFA	26	55	4	20	48	8
PUFA	30	57	3	20	64	11
Cholesterol	18	54	4	27	67	3
Carbohydrate	22	54	3	22	48	9

Sodium	35	66	3	30	67	3
Salt Eq	35	66	3	25	67	5
Potassium	26	71	3	23	69	2
Calcium	36	74	1	39	64	3
Magnesium	37	79	3	38	77	0
Phosphorus	37	65	1	31	67	5
Iron	25	61	4	34	67	0
Zinc	27	57	7	22	56	2
Copper	21	64	2	39	77	2
Manganese	22	55	5	27	58	5

Retinol	22	58	4	27	53	6
Retinol Eq	24	57	7	33	59	8
β-carotene Eq	29	60	2	23	59	5
Vitamin D	20	63	3	23	58	5
α-tocopherol	28	63	8	31	70	2
Vitamin K	32	68	4	44	73	3
Vitamin B_1_	27	60	5	28	55	5
Vitamin B_2_	27	73	1	31	66	3
Niacin	18	55	5	19	48	9
Vitamin B_6_	17	60	3	30	58	2
Vitamin B_12_	32	66	0	22	52	6
Folate	30	62	2	30	69	2
Pantothenic acid	34	70	3	34	56	3
Vitamin C	26	67	3	34	77	2

Total dietary fiber	24	59	3	36	70	6
Water soluble	22	62	8	30	59	5
Water insoluble	18	61	2	36	67	6

**Median**	**26**	**61**	**3**	**28**	**63**	**5**

24hDR, 24-hour dietary recall; Eq, equivalent; FFQ, food frequency questionnaire; MUFA, monounsaturated fatty acid; PUFA, polyunsaturated fatty acid; SFA, saturated fatty acid.

^a^Joint classification for energy intake was calculated using crude values.

Table 5 shows the degree of coincidence of food group intake. Median percentages were 25% for males and 28% for females for the ‘same category’, 65% for males and 64% for females for the ‘same or adjacent category’, and 3% for both males and females for the ‘completely opposite category’.

**Table 5.** Comparison of FFQ with 3-day non-consecutive 24hDR for energy-adjusted food groups based on joint classification by quintile (%)

	Male (*n* = 92)			Female (*n* = 64)		
	
	Same category	Same or adjacent category	Complete opposite category	Same category	Same or adjacent category	Complete opposite category
Cereals	25	65	2	30	64	0
Potatoes and starches	14	53	3	22	64	5
Sugar	22	65	7	16	47	9
Pulses	20	68	4	34	75	0
Nuts and seeds	28	64	3	14	47	6
Vegetables	30	67	3	33	64	3
Green and yellow vegetables	25	66	2	33	67	6
White vegetables	23	66	2	25	64	3
Fruits	30	65	1	31	77	2
Fungi	26	65	3	19	52	5
Algae	25	65	7	30	72	2
Fish and shellfish	29	63	2	28	67	5
Meats	23	54	7	22	47	5
Eggs	28	60	1	31	67	2
Milk and dairy products	41	82	0	41	77	2
Fats and oils	24	52	7	34	55	3
Confectioneries	27	58	2	28	63	6
Beverages	30	68	5	16	52	3
Seasonings and spices	25	70	5	20	59	5

**Median**	**25**	**65**	**3**	**28**	**64**	**3**

24hDR, 24-hour dietary recall; FFQ, food frequency questionnaire.

## DISCUSSION

In this study, we found that an FFQ developed and validated to estimate the food intake of middle-aged persons had comparable validity for young athletes. Although the median CCs were generally lower than those in a previous study,^11^ this FFQ might be a useful tool for assessing the habitual dietary intake of specific nutrients and food groups by collegiate athletes.

Consistent with the findings of Takachi et al,^11^ we found that under- and overestimation of food group intakes was more prevalent than that of nutrient intakes. Under- and overestimation was notably observed in both males and females for ‘sugar’, ‘seasonings and spices’, and ‘beverages’. Takachi et al^11^ also reported an 80% underestimation in ‘sugar’ and ‘seasonings and spices’. In the FFQ, questions regarding the estimation of ‘sugar’ referred only to sugar for coffee and tea; cooking sugar was not considered in the intake level. The intake of ‘seasonings and spices’ was calculated by adding the intake of specific seasonings (eg, salad dressing) and the estimated amount of cooking salt. In Japan, flavoring with sauces, such as soy sauce, is very common. A possible explanation for the underestimation of ‘sugar’ and ‘seasonings and spices’ might therefore be the lack of related food items, such as soy sauce. Although Takachi et al^11^ reported that beverages tended to be overestimated, our data show that they were underestimated by approximately 50%. During training, athletes frequently drink liquids to replace fluids. However, most of the portion sizes are fixed as small tea cups (120 mL per cup). In addition, sports drinks were not listed in the FFQ food items, which might have also affected the results. To understand the beverage intake of athletes, portion sizes need to be revised, and sports drinks commonly consumed by athletes need to be added to the list of beverage items.

In the present study, the degree of coincidence among quintile categories was comparable to that reported by Takachi et al.^11^ Based on these findings, we consider this FFQ to be suitable for categorizing individual nutrient and food group intakes in collegiate athletes. In both males and females, CCs exceeded 0.4 for the nutrients calcium and vitamin C and for the food groups of vegetables, fruits, and milk and dairy products. The relationship between these nutrients and food groups and the condition of athletes is currently being studied.^21^^–^^25^ Therefore, this FFQ might be useful in epidemiology studies that aim to identify a relationship between food intake and the condition of athletes. However, as total fat intake demonstrated markedly lower ranking validity in both males and females than in previous studies, results for total fat intake using this FFQ in intake and outcome-related epidemiology studies must be interpreted with caution. In 19 studies examining the validity of FFQs, the median CC for total fat intake was 0.46.^10^ In the present study, however, the CC for total fat intake was 0.18 for males and 0.22 for females. When an investigation was conducted to identify the food groups that affected total fat, the intake of food groups of ‘meats’ and ‘fats and oils’ were strongly associated with total fat intake (data not shown). These results might have been affected by measurement errors of these food groups by the FFQ, 24hDR, or both. Further, given that energy intake was calculated from the intakes of carbohydrates, proteins, and fats in the present study, the CC of energy intake was lower than in the previous study^11^ (markedly so in males). Errors in estimating the intake of ‘meat’ and ‘fats and oils’ might therefore also affect the CC of energy intake. Given that both the intake of ‘meat’ and of ‘fats and oils’ assessed by the FFQ were lower than those of the 24hDR, their portion sizes should be increased. In addition, given that the intake of ‘meat’ in the FFQ was estimated based on the type of meat and cooking pattern, the reduction in estimation errors and optimization of CCs for questions related to this intake might be effective. Similarly in females, CC of total fat declined from 0.22 to −0.08 after energy-adjustment. Although median CC typically increases when adjusted for energy intake, this is not necessarily the case for macronutrients.^11^^,^^26^^–^^30^ In other words, the decrease in CC after adjustment for energy, particularly in females, was likely due to either or both the FFQ or 24hDR’s larger measurement errors in energy intake than in total fat intake.^28^ Optimizing the estimation of the intake of ‘meat’ and ‘fats and oils’ is therefore also important for the accurate estimation of energy intake in females.

In the present study, we used a validated 24hDR to estimate the energy intake of collegiate athletes. Many validity studies of FFQs in Japan use a weighed DRM as a reference method.^10^ However, the weighed DRM increases the burden placed on participants, and measuring contents and amounts of dine-out or take-out food is difficult. Therefore, 24hDR has been used as a reference method in many studies, such as the National Nutrition Survey conducted by the United States Department of Agriculture, large-scale epidemiological studies of disease and diet,^15^^,^^16^ and studies targeting children, whose diets are difficult to record.^17^^,^^19^ The participants of the present study were college students, of whom more than 80% prepare their own meals. Considering the likelihood that participants frequently dined out or consumed take-out food, we used the 24hDR to lighten the burden on them. Six studies^31^^–^^36^ that compared energy intakes estimated from 24hDR and DRM reported that 24hDR tended to underestimate intake. In DRM studies in athletes, mean (standard deviation) energy intake was 2940 (556) kcal in male collegiate athletes by a weighed DRM^37^ and 1989 (466) kcal in female collegiate athletes by a DRM with food photographs.^38^ Mean energy intake from our present 24hDR was similar to intake in these previous studies, indicating that 24hDR is a valid method for assessing dietary intake in collegiate athletes.

Several limitations to the present study warrant mention. First, participants were not selected at random, potentially introducing selection bias. Second, as a dietary survey by FFQ could not be conducted prior to the first 24hDR, the reproducibility of the FFQ could not be assessed. Therefore, the FFQ still requires further investigation for validation of long-term intake, as was performed in the previous study.^11^ In addition, as the FFQ data were collected immediately after the third 24hDR, FFQ validation might have been overestimated due to information bias. Third, we used 24hDR as a reference method. Both 24hDRs and FFQs are prone to measurement error associated with recall bias and awareness of portion size. Errors associated with these methods are therefore not mutually independent, and the CCs might have been overestimated.^39^ However, we used the multiple-pass interview technique for 24hDRs to minimize measurement errors. Fourth, as our study was limited to a certain time period, it is not certain whether the FFQ captured seasonal and periodical changes in meals. However, FFQs are suitable for assessing habitual intake, and a DRM or 24hDR might be more suitable when the food environment drastically changes within a short time frame, such as immediately before games or during training camps.

### Conclusions

The validity of this FFQ in young athletes is comparable to that in middle-aged populations, for which it was originally developed. This FFQ might therefore be a useful tool for assessing habitual dietary intake in collegiate athletes, especially for the nutrients calcium and vitamin C and for the food groups of vegetables, fruits, and milk and dairy products. However, an FFQ with added or modified questions, particularly for sports drinks, meat, and fats and oils, may be more suitable for estimating dietary intake in collegiate athletes.

## ONLINE ONLY MATERIAL

Abstract in Japanese.
