# Supplementary material for: Validity of a Semi-Quantitative Food Frequency Questionnaire for Collegiate Athletes
Source: J Epidemiol. 2016 Jun 5;26(6):284–91. doi: 10.2188/jea.JE20150104 (PMC4884896; doi:10.2188/jea.JE20150104)
Supplement: Abstract in Japanese. [file je-26-284-s001.pdf]

大学生アスリートにおける半定量食物摂取頻度調査票の妥当性

Validity of a semi-quantitative food frequency questionnaire for collegiate athletes

砂見綾香[1,2]、佐々木和登[1]、鈴木良雄[3]、小熊伸英[3]、石原淳子[4]、中井あゆみ[1]、安田純[1]、横山友里[1,2]、吉﨑貴大[5]、多田由紀[6]、日田安寿美[6]、川野因[6]

[1] 東京農業大学大学院　農学研究科　食品栄養学専攻
[2] 日本学術振興会特別研究員
[3] 順天堂大学大学院　スポーツ健康科学研究科
[4] 相模女大学　栄養科学部　管理栄養学科
[5] 東洋大学　食環境科学部
[6] 東京農業大学　応用生物科学部　栄養科学科

要約

背景: 食物摂取頻度調査票（food frequency questionnaires、FFQ）は様々な集団を対象に開発され、妥当性が検証されている。しかし我々の知る限りでは、若年アスリートのためのFFQの妥当性はいまだ検証されていない。そこで我々は、中高年者の食事量を推定するために開発され、妥当性が検証されているFFQが若年アスリートの食事量推定においても妥当と認められるか否か、検討した。

目的: 本研究では「多目的コホートに基づくがん予防など健康の維持・増進に役立つエビデンスの構築に関する研究」のために開発されたFFQを、思い出し期間を変更して用いた。156名（うち、男性92名）がFFQおよび不連続3日間の24時間思い出し食事調査（24-hour dietary recall　、24hDR）を完了した。平均値推定の妥当性は、24hDRとFFQの%差を用いて評価した。順位推定の妥当性には、スピアマンの順位相関係数（correlation coefficient、CC）および五分位の同時分類による一致度を用いた。

結果:男女ともに、FFQはエネルギー摂取量をおよそ10%過小評価した。栄養素35項目では、deattenuated CCの中央値（範囲）が男性で0.30（0.10から0.57）、女性で0.32（-0.08から0.62）であった。食品群19項目では、男性で0.32（0.17から0.72）、女性で0.34（-0.11から0.58）であった。栄養素および食品群で正反対の分位に分類された割合は3%から5%であった。

結論:中高年者の食事量推定のために開発され、妥当と評価されたFFQは、若年アスリートにおいても同等の妥当性を示した。今回のFFQは大学アスリートの習慣的食事量を評価するには有用で、特に栄養素ではカルシウム、ビタミンC、食品群では野菜類、果実類、牛乳・乳製品の評価に優れているだろう。

キーワード: 食事調査、アスリート、FFQ、24時間思い出し法
